# New species and records of the stonefly genus *Neoperla* (Plecoptera, Perlidae) from Jinhuacha Nature Reserve, Guangxi of China

**DOI:** 10.3897/zookeys.351.6259

**Published:** 2013-11-15

**Authors:** Guo–Quan Wang, Wei–Hai Li, Juan Yang

**Affiliations:** 1 Department of Plant Protection, Guangxi University, Nanning, Guangxi 530004, China; 2 Department of Plant Protection, Henan Institute of Science and Technology, Xinxiang, Henan 453003, China

**Keywords:** Plecoptera, Perlidae, *Neoperla mesospina*, *Neoperla latispina*, new species, China

## Abstract

Two new *Neoperla* species (*Neoperla mesospina*, *Neoperla latispina*) are described from the adult male stage from the Jinhuacha Nature Reserve, Guangxi of China. The new species are compared with similar taxa. Taxonomic remarks are also provided for *N. transversprojecta* Du & Sivec and *N. yao* Stark. The latter species is newly recorded for Guangxi.

## Introduction

The stonefly genus *Neoperla* is a species-rich perlid stonefly genus in China (Sivec et al. 1988, DeWalt et al. 2013). The works of this genus in China was mainly contributed by Chu (1929), Du (1999, 2000a, 2000b), Du and Sivec (2004, 2005), Du and Wang (2005, 2007), Du et al. (1999, 2001), Li et al. (2011a), Li et al. (2011b), Li and Wang (2011), Li et al. (2012a), Li et al. (2012b), Li and Li (2013), Li et al. (2013a), Li et al. (2013b), Qin et al. (2013), Sivec and Zwick (1987), Wang et al. (2013),Wu (1935, 1938, 1948, 1962, 1973), Wu and Claassen (1934), Yang and Yang (1990, 1991), and Yang and Yang (1992, 1993, 1995a, 1995b, 1996, 1998).

In the present paper, four species ofthe genus *Neoperla* are threated from the specimens collected in Jinhuacha Nature Reserve, Guangxi of China in the recent two years, including two new one: *Neoperla latispina* sp. novand *Neoperla mesospina* sp. n. The Reserve is located in Fangchenggang city of Guangxi Zhuang Autonomous Region and was established to protect rare or endangered species of *Camellia nitidissima* Chi (Chinese name Jinhuacha).

## Material and methods

The specimens used in this study were collected by light trap. Types and other examined material are deposited in the Insect Collection of Henan Institute of Science and Technology (HIST), Xinxiang, and the Entomological Museum of China Agricultural University (CAU), Beijing. They were examined with the aid of a Motic SMZ 168 microscope and the color illustrations were captured using digitalized software Motic Images Advanced 3.2. All specimens were kept in 75% ethanol. Aedeagi were everted using the cold maceration technique of Zwick (1983). Terminology follows that of Sivec et al. (1988). All the scale lines in the figures stand for 0.5 mm.

## Results

### Neoperla latispina

Wang & Li sp. n.

http://zoobank.org/96D00CD1-528F-40F4-891A-0B5B9CF19762

http://species-id.net/wiki/Neoperla_latispina

Figs 1 –2

#### Type material.

1 male (CAU), China: Guangxi, Fangcheng, Jinhuacha Nature Reserve, 21°76,09'N, 108°43,49'E, light trap, 15 May 2013, G.Q. Wang.

#### Male.

**Figure 1.**
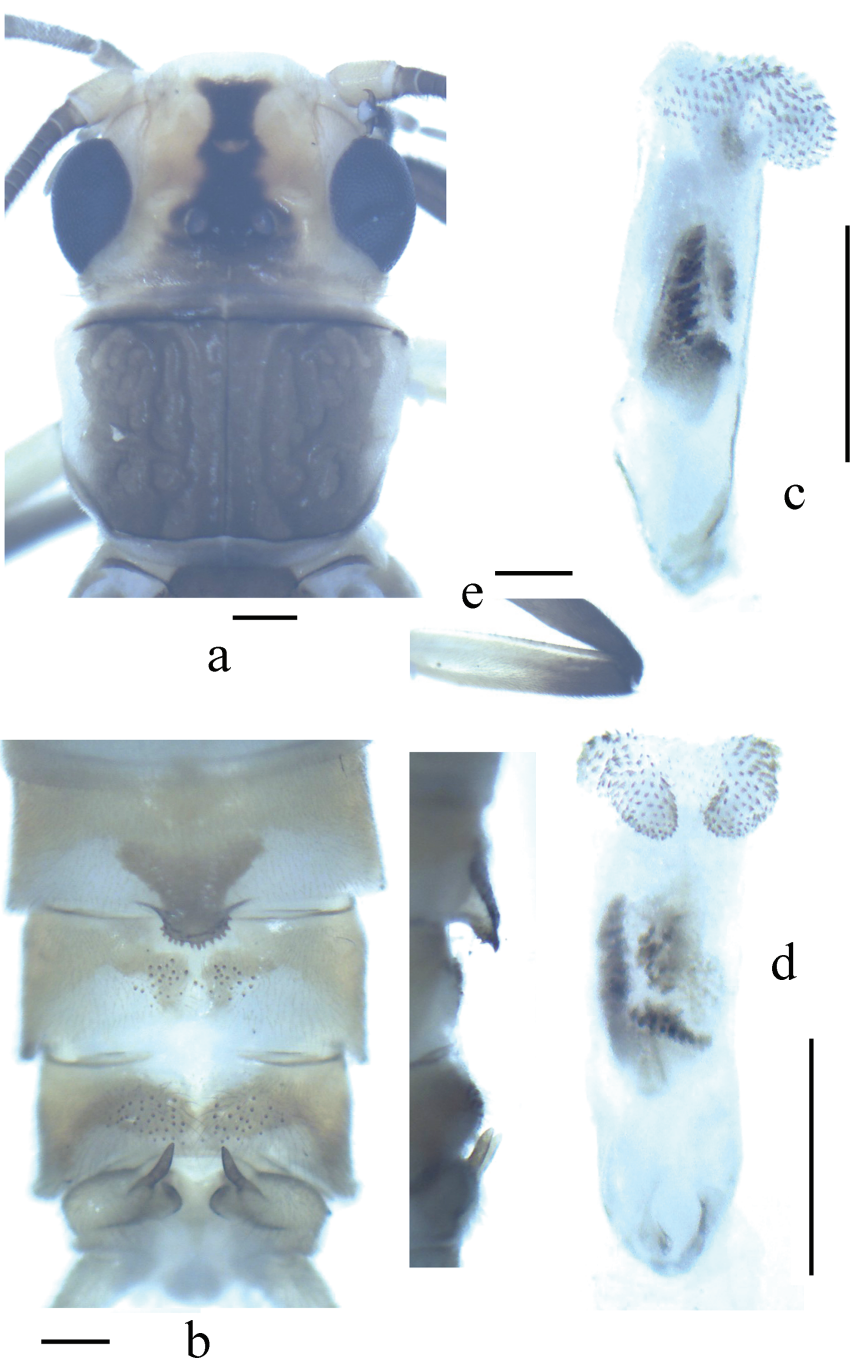
*Neoperla latispina* Wang & Li, sp. n. Male. **a** Head and pronotum, dorsal view **b** Terminalia, dorsal and lateral views **c** Aedeagus before eversion, lateral view **d** Aedeagus before eversion, dorsal view **e** foreleg femur.

Forewing length 12.6 mm. Distance between ocelli about 1.5× as wide as diameter of the ocellus. Head pale yellow, slightly wider than pronotum, with a black area covering ocelli which extends forward to contacting a quadrate black stigma on frons (Fig. 1a); compound eyes black and antennae dark except scape pale; maxillary palpi brownish. Pronotum with obscure rugosities and pale lateral margins, meso- and metathorax mostly brown (Fig. 1a); wing membrane subhyaline, veins brown; legs dark brown with femora pale brown, but distal fourth of foreleg femora dark brown (Fig. 1e).

**Figure 2.**
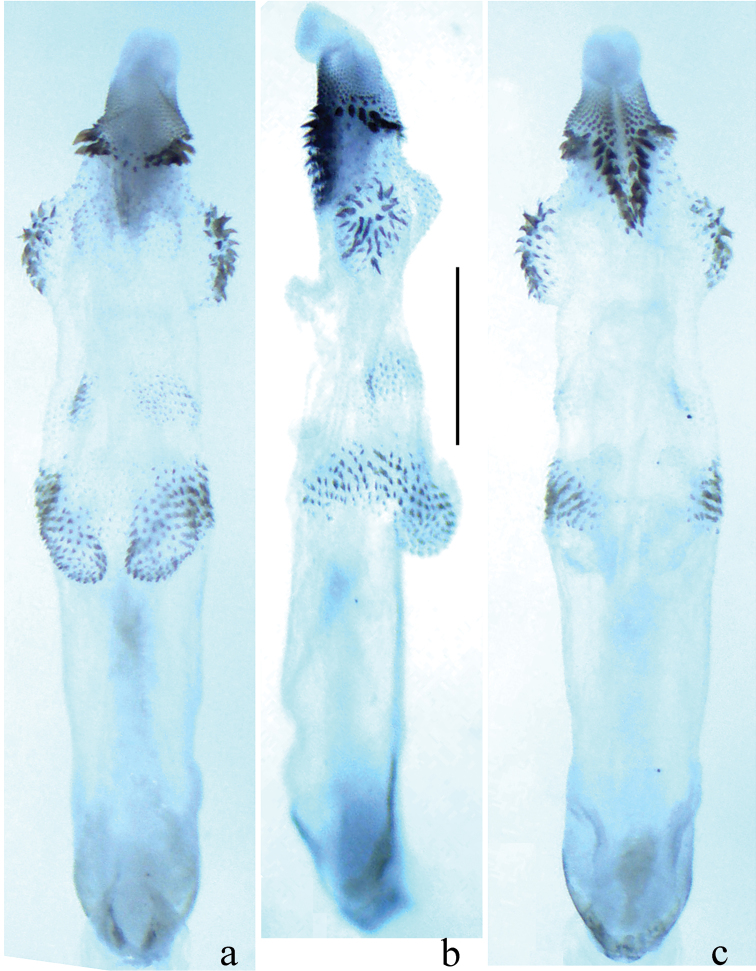
*Neoperla latispina* Wang & Li, sp. n. Male. **a** Aedeagus, dorsal view **b** Aedeagus, lateral view **c** Aedeagus, ventral view.

*Terminalia*. The posterior margin of tergum 7 with a trapezoidal sclerotized area extended into a rounded quadrate process covered with distal sensilla basiconica (Fig. 1b). Tergum 8 with two median weak humps covered by sensilla basiconica (Figs 1b). Tergum 9 with two patches of sensilla basiconica. Hemitergal processes of tergum 10 sclerotized, with rod-like base and sharp apex (Fig. 1b). Aedeagal tube membranous with a weak basodorsal sclerite, apically with a pair of separate dorsal spinous lobes covered by small spines connected with two lateral spinous bands, in lateral view the lobes plump and nipple like (Figs 1c–d, 2). Aedeagal sac slightly longer than tube, essentially straight along with tube in outline; two spinulose dorsal patches present at slightly swollen base, single ventral lobe without spines, located near midlength of sac, a pair of mesolateral protrusions rounded, covered by large spines and two nearby elevated dorsal lobes triangular in lateral view covered with tiny spines; distal portion of sac with two rows of large spines which extend laterally below the apical fine spinules (Fig. 2).

#### Female.

Unknown.

#### Etymology.

The specific epithet refers to the lateral spinous patch of aedeagal sac.

#### Distribution.

China (Guangxi).

#### Diagnosis and remarks.

*Neoperla latispina* belongs to *oculata* species complex defined by Zwick (1986) in the *montivaga* species group (Zwick 1983) which includes species bearing a similar T7 lobe and several lobes, protrusions or finger shaped extensions of the aedeagal sac (see figs 1–2 and figs 51–63 in Zwick 1986 for comparison). This species shares dorsal lobe characteristics of the aedeagal tube with *Neoperla securifera* Zwick 1986 and *Neoperla multilobata* Zwick 1986 (figs 57 & 59 in Zwick 1986). *Neoperla latispina* also shares a straight outline of the extruded aedeagus with *Neoperla multilobata* whereas the sac of *Neoperla securifera* stands at right angle to tube. However, the dorsal lobes of aedeagal tubein *Neoperla latispina* are paired but that of *Neoperla multilobata* is single. Additionally, *Neoperla latispina* bears only single ventral lobe near midlength while *Neoperla multilobata* has spinous ventrobasal and ventrodistal lobes (fig. 59 in Zwick 1986).

### Neoperla mesospina

Li & Wang sp. n.

http://zoobank.org/1D766031-1F27-48B8-B91A-C1D12FD2D661

http://species-id.net/wiki/Neoperla_mesospina

Figs 3 –4

#### Type material.

Holotype: 1 male (HIST), Guangxi, Fangcheng, Jinhuacha Nature Reserve, 21°76,09'N, 108°43,49'E, light trap, 16 April 2012, G.Q. Wang. Paratypes:2 males (HIST), same as holotype; 1 male (CAU), same locality, 2013. May 15, G.Q. Wang.

#### Male.

**Figure 3.**
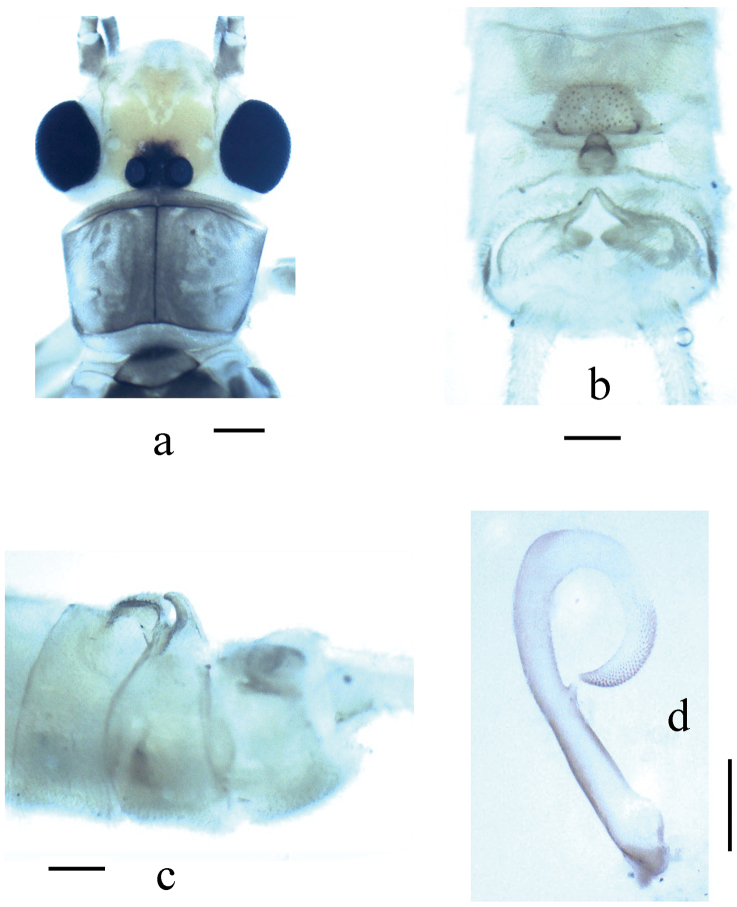
*Neoperla mesospina* Li and Wang, sp. n. Male. **a** Head and pronotum, dorsal view **b** Terminalia, dorsal view **c** Terminalia, lateral view **d** Aedeagus, lateral view.

Forewing length 11.1–11.3 mm. Distance between ocelli about as wide as diameter of the ocellus. Head mostly yellow brown, lateral margins and frons pale, a subquadrate dark area covering ocelli, slightly wider than pronotum; antennae brown; compound eyes dark; mouthparts brown (Fig. 3a). Thorax brownish with darker median stripe and scattered rugosities, legs brown; wings pale. Abdomen brownish yellow.

**Figure 4.**
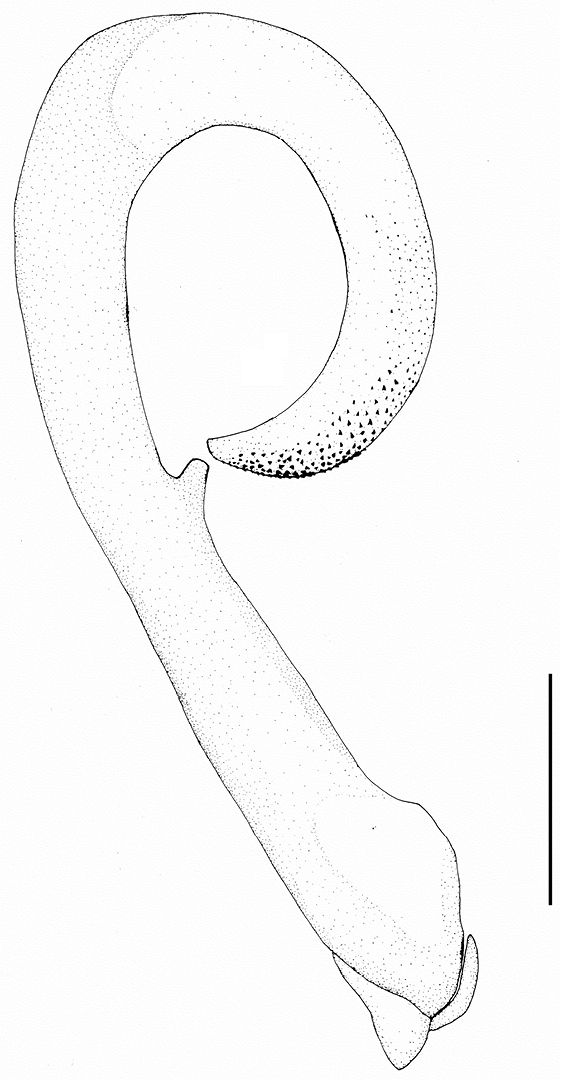
*Neoperla mesospina* Li and Wang, sp. n. Male aedeagus, lateral view.

*Terminalia*. Tergum 7 with an elevated trapezoidal median process at posterior margin, which is covered by many tiny sensilla basiconica. Process of tergum 8 moderately sclerotized, recurved backward and tougue–shaped, lateral marings with tiny spines. Tergum 9 without sensilla patches. Hemitergal processes of tergum 10 strongly sclerotized and with slightly curved apex (Figs 3a, b). Aedeagal tube long and slender, relatively straight and moderately sclerotized, medially with a nipple-like process in ventral surface; everted sac strongly curved ventrad as a loop, about half as long as tube, irregular rows of small to median sized spines present along dorsal and lateral surfaces from medial portion to apex of the sac (Figs 3d & 4).

#### Female.

Unknown.

#### Etymology.

The specific epithet refers to the location of the nipple-like process on the aedeagal tube.

#### Distribution.

China (Guangxi).

#### Diagnosis and remarks.

The new species seems closely related to *Neoperla dao* Stark & Sivec, 2008, a recently described species from Vinh Phu Province of Vietnam. They are very similar in head pattern, general features of male terminalia and the aedeagal tube. However, the new species can be easily separated from *Neoperla dao* Stark & Sivec by the relatively long (about half as long as tube) and strongly curved aedeagal sac in lateral view. In *Neoperla dao* Stark & Sivec, the sac is very short, somewhat straight and triangular in outline (fig. 22 in Stark and Sivec 2008). There are no other Chinese species of *Neoperla* that appear related to *Neoperla mesospina*.

### Neoperla transversprojecta

Du & Sivec, 2004

http://species-id.net/wiki/Neoperla_transversprojecta

Fig. 5

Neoperla transversprojecta Du & Sivec, 2004. In Yang X-K (Ed) Insects from Mt. Shiwandashan Area of Guangxi. China Forestry Publishing House: 42. Type locality: China, Guangxi, Fangcheng County, Banba town; Li et al. 2011. Zootaxa 2735: 57.

#### Material examined.

2 males (HIST), China: Guangxi, Fangcheng, Jinhuacha Nature Reserve, 21°76,09'N, 108°43,49'E, light trap, 2013. May 15, G.Q. Wang.

#### Distribution.

China (Guangxi).

#### Remarks.

This species was originally described by Du and Sivec (2004). 2 males were available to the present study and only the tip of sac shows a slight difference with original drawing (fig. 4 in Du and Sivec 2004). The dorsal patch of subapical spines in the present material seems prominent than as in original drawing.

**Figure 5.**
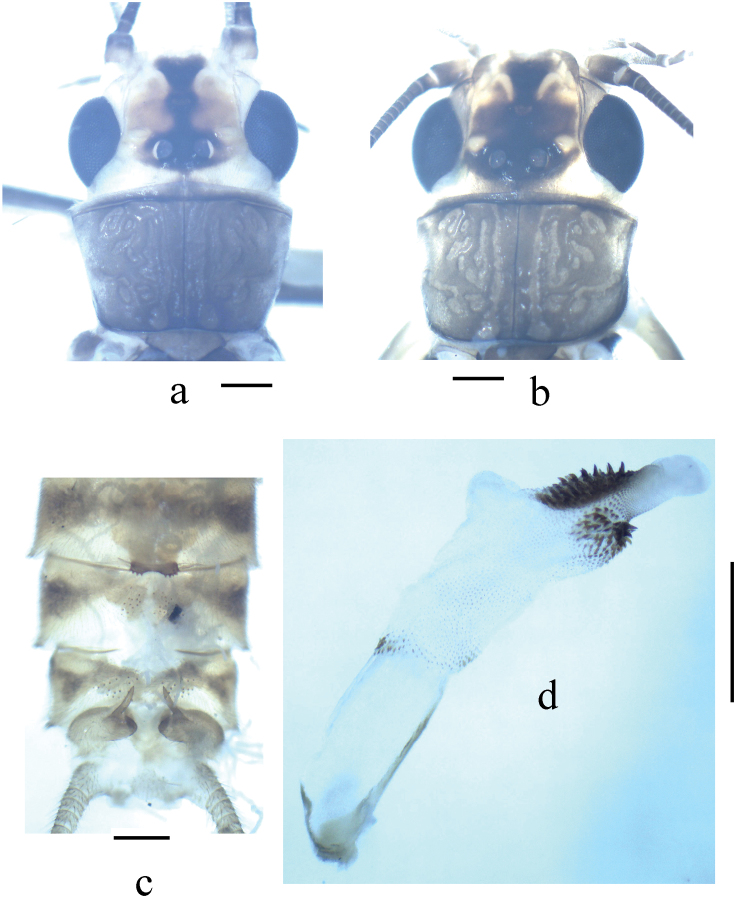
*Neoperla transversprojecta* Du & Sivec, 2004. Male. **a** Head and pronotum (teneral specimen), dorsal view **b** Head and pronotum (older specimen), dorsal view **c** Terminalia, dorsal view **d** Aedeagus, lateral view.

### Neoperla yao

Stark, 1987

http://species-id.net/wiki/Neoperla_yao

Fig. 6

Neoperla yao Stark, 1987. Aquatic Insects 9: 47. Type locality: Vietnam, 6 km S Dalat; Stark & Sivec, 2008. Illiesia 4: 41; Wang et al. 2013. Zookeys 313: 87.

#### Material examined.

1 male (HIST), China: Guangxi, Fangcheng, Jinhuacha Nature Reserve, 21°76,09'N, 108°43,49'E, light trap, 2012. April 16, G.Q. Wang.

#### Distribution.

Vietnam, China (Guangxi, Guangdong).

#### Remarks.

This species was originally described by Stark (1987) from Vietnam and China based on three males (two from Vietnam, one from China), its female was recently associated (Stark and Sivec 2008). One male was available to the present study and the tip of sac seems slightly hooked with sharper apex that appears somewhat blunt in original drawing (fig. 6 in Stark 1987).

**Figure 6.**
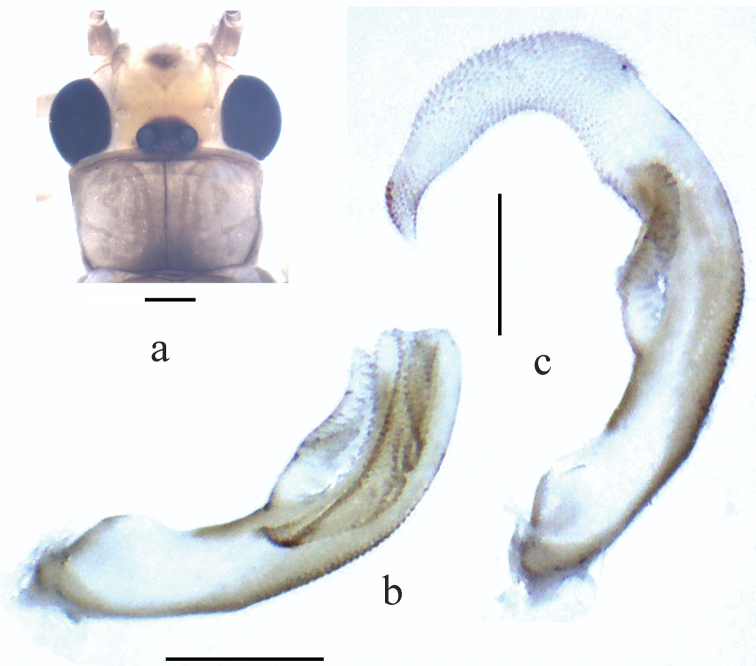
*Neoperla yao* Stark, 1987. Male. **a** Head and pronotum (teneral specimen), dorsal view **b** Aedeagus before eversion, lateral view **c** Aedeagus, lateral view.

## Concluding remarks

Previous studies on the genus *Neoperla* from Guangxi include Wu (1948), who described *Neoperla curvispina* and *Neoperla rotunda* from Mountain Yaoshan. Subsequently Yang and Yang (1990) described *Neoperla wui* from Jinxiu, and Du (1998) reported the presence of *Neoperla mnong* Stark, 1987 from Guangxi. Du and Sivec (2004) summarized the stonefly fauna from Mountain Shiwandashan in Guangxi, describing three new *Neoperla* species and adding two other records. Recently, two new species were added from Guangxi by Li et al. (2013b) and Wang et al. (2013). However, there was still no record of the genus *Neoperla* or other stoneflies in previous studies on the insect fauna of the Jinhuacha Nature Reserve. In this study, two additional new species are described and a new record for Guangxi is recorded. Therefore, there are up to 14 known *Neoperla* species from Guangxi presently.

## Supplementary Material

XML Treatment for Neoperla latispina

XML Treatment for Neoperla mesospina

XML Treatment for Neoperla transversprojecta

XML Treatment for Neoperla yao
